# Effects of carnitine and its congeners on eicosanoid discharge from rat cells: implications for release of TNFα

**DOI:** 10.1155/S0962935193000778

**Published:** 1993

**Authors:** Ingrid M. Garrelds, Graham R. Elliott, Wanda M. Pruimboom, Freek J. Zijlstra, Iván L. Bonta

**Affiliations:** 1 Department of Pharmacology Faculty of Medicine Erasmus University Rotterdam P.O. Box 1738 Rotterdam 3000 DR The Netherlands; 2 Department of Pharmacology M.B.L. P.O. Box 45 Rijswijk 2280 AA The Netherlands

## Abstract

THE acyl carrier coenzyme A (CoA) is involved in fatty acid metabolism. The carnitine/CoA ratio is of particular importance in regulating the transport of long-chain fatty acids into mitochondria for oxidation. Also CoA has a role in the formation and breakdown of products from both the cyclooxygenase and lipoxygenase pathways of the precursor arachidonic acid. In the present study the effect of 4 days feeding of 300 mg/kg/day of L-carnitine, acetyl Lcarnitine and propionyl L-carnitine on the basal and calcium ionophore (A23187) stimulated release of arachidonic acid metabolites from rat carrageenin elicited peritoneal cells was investigated. There were two series of experiments carried out. In the first, the harvested peritoneal cell population consisted of less than 90% macrophages and additional polymorphonuclear (PMN) leucocytes. The basal release of prostaglandin E_2_ (PGE_2_), 6-ketoprostaglandin F_1α_ (6-keto-PGF_1α_) and leukotriene B_4_ (LTB_4_) was stimulated by all treatments. The A23187 stimulated release of 6-keto-PGF_1α_ and LTB_4_ was increased by all three compounds. The 6-keto-PGF_1α_:TxB_2_ and 6-keto-PGF_1α_:LTB_4_ ratios were increased by carnitine treatment. These results suggested that carnitine could modify the macrophage component of an inflammatory site *in vivo*. In the second series of experiments the harvested cell population was highly purified (>95% macrophages) and none of the compounds fed to the rats caused a change of either eicosanoid or TNFα formation. Moreover the 6-keto-PGF_1α_:TxB_2_ and 6-keto-PGF_1α_:LTB_4_ ratios were not enhanced by any of the compounds tested. It is conceivable that in the first series the increased ratios 6-keto-PGF_1α_:TxB_2_ and 6-keto-PGF_1α_:LTB_4_ reflected the effect of carnitine or its congeners on PMN leucocytes rather than on macrophages.


Mediators of Inflammation 2, S57–S62 (1993)

### Effects of carnitine and its congeners on eicosanoid discharge from rat cells: implications for release of TNFα

Ingrid M. Garrelds,[1,CA] Graham R. Elliott,[2] Wanda M. Pruimboom,[1] Freek J. Zijlstra[1] and Iván L. Bonta[1]

[1]Department of Pharmacology, Faculty of Medicine, Erasmus University Rotterdam, P.O. Box 1738, 3000 DR Rotterdam, The Netherlands; [2]Department of Pharmacology, M.B.L. P.O. Box 45, 2280 AA Rijswijk, The Netherlands

[CA] Corresponding Author

**THE acyl carrier coenzyme A (CoA) is involved in fatty acid metabolism. The carnitine/CoA ratio is of particular importance in regulating the transport of long-chain fatty acids into mitochondria for oxidation. Also CoA has a role in the formation and breakdown of products from both the cyclooxygenase and lipoxygenase pathways of the precursor arachidonic acid. In the present study the effect of 4 days feeding of 300 mg/kg/day of L-carnitine, acetyl L-carnitine and propionyl L-carnitine on the basal and calcium ionophore (A23187) stimulated release of arachidonic acid metabolites from rat carrageenin elicited peritoneal cells was investigated. There were two series of experiments carried out. In the first, the harvested peritoneal cell population consisted of less than 90% macrophages and additional polymorphonuclear (PMN) leucocytes. The basal release of prostaglandin $E_2$ ($PGE_2$), 6-keto-prostaglandin $F_{1\alpha}$ (6-keto-$PGF_{1\alpha}$) and leukotriene $B_4$ ($LTB_4$) was stimulated by all treatments. The A23187 stimulated release of 6-keto-$PGF_{1\alpha}$ and $LTB_4$ was increased by all three compounds. The 6-keto-$PGF_{1\alpha}$:$TxB_2$ and 6-keto-$PGF_{1\alpha}$:$LTB_4$ ratios were increased by carnitine treatment. These results suggested that carnitine could modify the macrophage component of an inflammatory site *in vivo*. In the second series of experiments the harvested cell population was highly purified (>95% macrophages) and none of the compounds fed to the rats caused a change of either eicosanoid or TNFα formation. Moreover the 6-keto-$PGF_{1\alpha}$:$TxB_2$ and 6-keto-$PGF_{1\alpha}$:$LTB_4$ ratios were not enhanced by any of the compounds tested. It is conceivable that in the first series the increased ratios 6-keto-$PGF_{1\alpha}$:$TxB_2$ and 6-keto-$PGF_{1\alpha}$:$LTB_4$ reflected the effect of carnitine or its congeners on PMN leucocytes rather than on macrophages.**

**Key words:** Eicosanoids, Polymorphonuclear leucocytes, Rat peritoneal macrophages, Tumour necrosis factor α

### Introduction

Fatty acids are oxidized in mitochondria. They are activated before they enter the mitochondrial matrix. Adenosine triphosphate (ATP) drives the formation of a thioester linkage between the carboxyl group of a fatty acid and sulphydryl group of coenzyme A (CoA). This reaction occurs on the outer mitochondrial membrane, where it is catalysed by acyl CoA synthetase (also called fatty acid thiokinase). The activation of a fatty acid occurs in two steps. First, the fatty acid reacts with ATP to form an acyl adenylate. In this mixed anhydride, the carboxyl group of a fatty acid is bonded to the phosphoryl group of adenosine triphosphate (AMP). The sulphydryl group of CoA then attacks the acyl adenylate, which is tightly bound to the enzyme, to form acyl CoA and AMP.

Fatty acids are activated on the outer mitochondrial membrane, whereas they are oxidized in the mitochondrial matrix (Fig. 1). Long-chain acyl CoA molecules do not readily transverse the inner mitochondrial membrane, and so a special transport mechanism is needed. Activated long-chain fatty acids are carried across the inner mitochondrial membrane by L-carnitine (β-hydroxy-(τ-N-trimethylammonio)-butyrate), a zwitterionic compound formed from lysine. The acyl group is transferred from the sulphur atom of CoA to the hydroxyl group of carnitine to form acyl carnitine. This reaction is catalysed by carnitine acyltransferase I, which is located on the cytosolic face of the inner mitochondrial membrane. Acyl carnitine is then shuttled across the inner mitochondrial membrane by a translocase. The acyl group is transferred back to CoA on the matrix side of the membrane. This reaction is catalysed by acyltransferase II.[1–3]

CoA also has a role in the formation and breakdown of products from both the cyclooxygenase and lipoxygenase pathways of the fatty

© 1993 Rapid Communications of Oxford Ltd

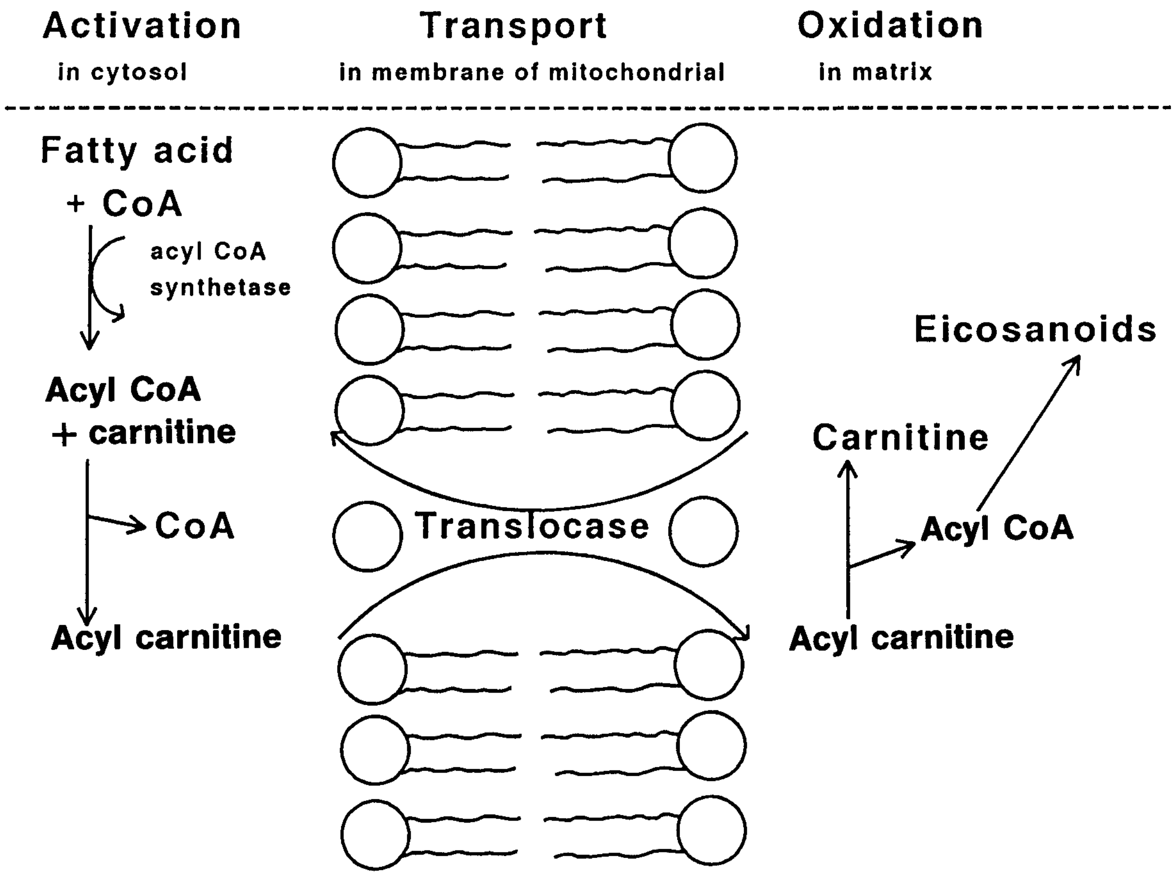

FIG. 1. Schematic representation of the activation of long-chain fatty acids in the cytosol, the entry of acyl carnitine into the mitochondrial matrix mediated by a translocase and the oxidation of acyl CoA in eicosanoids in the mitochondrial matrix.

acid, and arachidonic acid metabolism.[4] Recently the oxidation of the cyclooxygenase product prostaglandin $E_2$ ($PGE_2$) in rat liver peroxisomes and mitochondria was shown to require the activation to $PGE_2$–CoA by microsomal $PGE_2$–CoA synthetase.[5] $PGE_2$ is recognized as an important inhibitor of cell migration and of mediator formation at inflammatory sites. Enhanced formation of cyclic AMP is involved in these inhibitory effects. Similar inhibitory effects may be exerted by prostacyclin ($PGI_2$), which also enhances the intracellular levels of cyclic AMP. The lipoxygenase metabolite leukotriene $B_4$ ($LTB_4$) is degraded through omega- and beta-oxidation in neutrophils and liver cells. The formation of $LTB_4$–CoA esters which occurs in rat liver microsomes may be essential for $LTB_4$ beta-oxidation.[6] $LTB_4$ is a potent chemotactic agent and promotes the formation of mediators at inflammatory sites. The degradation products of $LTB_4$ are less potent than the parent compound. Hence the formation of $LTB_4$–CoA esters and their subsequent beta-oxidation might be of importance in limiting $LTB_4$ activity at the sites of inflammation. Macrophages secrete a large variety of cytokines, such as interleukines and tumour necrosis factor $\alpha$ (TNF$\alpha$). In normal human alveolar macrophages, when stimulated for 24 h with lipopolysaccharide (LPS), the cyclooxygenase pathway was stimulated specifically.[7] A high $PGE_2$ concentration inhibits TNF$\alpha$ by increased levels of cyclic AMP. A low $PGE_2$ augments a high TNF$\alpha$ production, which is stimulated by guanylate cyclase.[8,9] Carnitine is known to enhance the formation of arachidonic acid from linoleic acid by isolated hepatocytes, since long-chain acyl carnitine can remove the inhibitory effect of long-chain acyl CoA on acetyl CoA carboxylase, a regulating enzyme in the fatty acid synthesis.[10] The serum carnitine and leucine levels were significantly decreased in patients on continuous ambulatory peritoneal dialysis (CAPD) for more than 4 months compared with levels of controls. This data suggested that malnutrition plays a role in the decrease of serum carnitine levels in patients receiving CAPD.[11] L-Carnitine has several beneficial effects on ischaemic heart diseases and arrhythmias in humans, where the drug augments the ischaemic heart tolerance to stress.[12] Carnitine deficiency can be defined as a decrease of intracellular carnitine, leading to an accumulation of acyl CoA esters and an inhibition of acyl transport via the mitochondrial inner membrane. Inhibition of the mitochondrial oxidation of long-chain fatty acids during fasting causes heart or liver failure. Patients with cardiomyopathy due to carnitine loss are improved by carnitine supplementation.[13–15]

In the present study on the role of carnitine in the eicosanoid and cytokine production at inflammatory sites, the eicosanoids formed from basal and A23187 stimulated, and TNF$\alpha$ formed from basal and LPS stimulated carrageenin induced peritoneal cells of rats after feeding L-carnitine, acetyl L-carnitine (formed during $\beta$-oxidation of even-chain fatty acids) and propionyl L-carnitine (formed during $\beta$-oxidation of uneven-chain fatty acids) were determined.

### Materials and Methods

*Animals and treatment:* Male Wistar rats were given 300 mg/kg carnitine or carnitine equivalent (acetyl carnitine and propionyl carnitine) (gifts of Sigma-Tau, Italy) dissolved in 1 ml distilled water, by intubation on days 1–4. Control animals were given distilled water. All animals were injected with 2 ml of a carrageenin (Marine Colloids Inc., USA) solution (1 mg/ml) intraperitoneally on day 1.

*Isolation/incubation of peritoneal cells:* Two series of experiments were carried out. In the first series (with neutrophil contamination >10%) on day 4, 1 h after the last administration of carnitine(s), the cells were isolated from pooled Gey's balanced salt solution washes of the peritonea of the rats (four rats/group) by density gradient centrifugation over Lymphoprep (Nyegaard Diagnostica, Norway) and suspended in Dulbecco's modification of Eagle's medium (DMEM) ($2 \times 10^6$ nucleated cells). The harvested peritoneal cell population consisted of less than 90% macrophages and >10% PMN leucocytes. Portions (1 ml) of suspension were incubated at 37°C for either 2 h (basal release) or 30 min with $10^{-6}$ M A23187 (ionophore stimulated release). The cells were then centrifugated and the supernatant fractions analysed for production of leukotriene $B_4$ ($LTB_4$), prostaglandin $E_2$ ($PGE_2$), thromboxane $B_2$ ($TxB_2$) and 6-keto-prostaglandin $F_{1\alpha}$ (6-keto-$PGF_{1\alpha}$) by radioimmunoassays.[16,17]

In the second series of experiments (without neutrophil contamination) on day 4, 1 h after the last administration of carnitine(s), the cells were obtained from each separate rat (nine rats/group) by washing the peritoneal cavity with 2 × 20 ml of phosphate buffered saline (PBS) (Oxoid, UK). The macrophages were isolated by density gradient centrifugation with Lymphoprep (Nycomed, Norway) and suspended in Dulbecco's modification of Eagle's medium (DMEM) (Life Technologies Ltd, UK) ($2 \times 10^6$ macrophages/ml). The harvested cell population consisted of >95% macrophages, approximately 3% PMN leucocytes and 2% other cells (lymphocytes, erythrocytes). Portions (1 ml) of suspension were incubated at 37°C for 30 min with or without $10^{-6}$ M A23187 (Sigma, USA) (ionophore stimulated release) and 24 h with or without 5 $\mu$g/ml LPS. The cells were then centrifugated and the supernatant fractions analysed for production of $LTB_4$, $PGE_2$, $TxB_2$ and 6-keto-$PGF_{1\alpha}$ by radioimmunoassays (basal and A23187 stimulated), and TNF$\alpha$ by MTT-tetrazolium bioassay (24 h with or without LPS).[17,18]

*Statistical analysis:* In the first series of experiments the values are given as the mean for each point ± SEM of three experiments. In the second series, the results are expressed as the mean ± SEM of nine experiments. Statistical significance was calculated using the two-tailed Mann-Whitney *U* test.

### Results

*Effect of feeding of carnitine or its congeners on the number of carrageenin induced peritoneal cells:* In the first series (with neutrophil contamination) of experiments all three compounds significantly reduced, by about half, the number of nucleated cells isolated from peritonea 4 d after an intraperitoneal injection of carrageenin ($\times 10^6 \pm$ SEM per rat): control 13 ± 2, carnitine 6 ± 1; acetyl carnitine 5 ± 1, propionyl carnitine 6 ± 0.6.[16]

In the second series (without neutrophil contamination) of experiments none of the compounds fed to the rats caused a decrease in the number of macrophages accumulated in the peritoneal cavity ($\times 10^6 \pm$ SEM per rat): control, 3.6 ± 0.5, carnitine, 4.4 ± 0.6, acetyl carnitine, 3.3 ± 0.4, propionyl carnitine, 4.6 ± 0.4.

*Effect of feeding of carnitine or its congeners on the basal and A23187 stimulated release of eicosanoids from carrageenin induced peritoneal cells:* In the first series (with neutrophil contamination) of experiments the basal release of $PGE_2$, 6-keto-$PGF_{1\alpha}$ and $LTB_4$ was stimulated by all treatments. In contrast, $TxB_2$ production was inhibited by feeding carnitine and acetyl carnitine or not modified by feeding propionyl carnitine (Fig. 2, Table 1). A23187 stimulated synthesis of 6-keto-$PGF_{1\alpha}$ and $LTB_4$ was further enhanced by all three compounds and acetyl carnitine and propionyl carnitine treatments increased the formation of $TxB_2$. However, no effects on $PGE_2$ formation were detected (Fig. 3, Table 1). The 6-keto-$PGF_{1\alpha}$:$TxB_2$ ratio, calculated from the basal and A23187 stimulated values, was increased by carnitine treatment (Table 2). In the presence of A23187 there was also an increase in the 6-keto-$PGF_{1\alpha}$:$LTB_4$ ratio (Table 2).[16] In the second

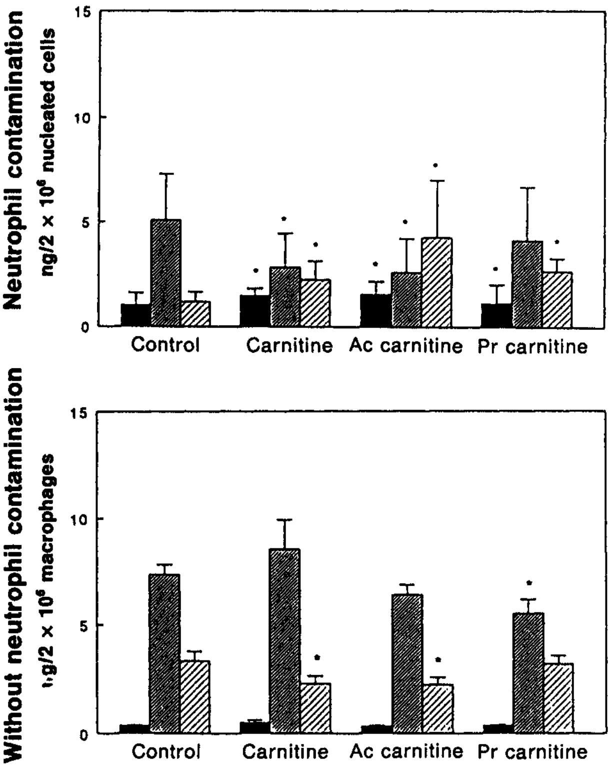

FIG. 2. Effect of feeding of carnitine or its congeners on the basal release of prostaglandin $E_2$ (■), thromboxane $B_2$ (▩) and 6-keto-prostaglandin $F_{1\alpha}$ (▨) from carrageenin induced peritoneal cells with or without neutrophil contamination. Statistical significance to the control group is shown as $^*p < 0.05$ according to Mann–Whitney $U$ test.

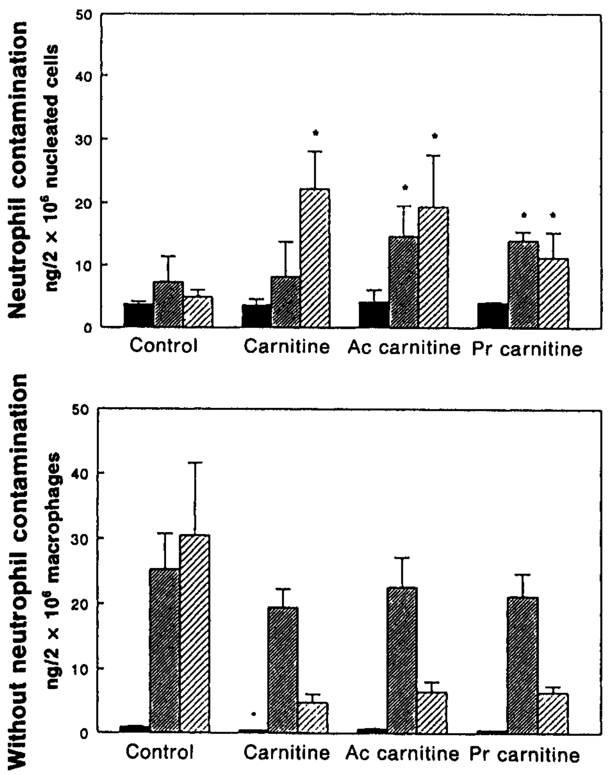

FIG. 3. Effect of feeding of carnitine or its congeners on the A23187 stimulated release of prostaglandin $E_2$ (■), thromboxane $B_2$ (▩) and 6-keto-prostaglandin $F_{1\alpha}$ (▨) from carrageenin induced peritoneal cells with or without neutrophil contamination. Statistical significance to the control group is shown as $^*p < 0.05$ according to Mann–Whitney $U$ test.

series (without neutrophil contamination) of experiments the basal and the A23187 stimulated release of $PGE_2$, $TxB_2$, 6-keto-$PGF_{1\alpha}$ and $LTB_4$ were not increased (Figs 2 and 3, Table 1).

The 6-keto-$PGF_{1\alpha}$:$TxB_2$ and the 6-keto-$PGF_{1\alpha}$:$LTB_4$ ratios were not enhanced by any of the compounds tested (Table 2).

### Discussion

It was recently shown that in a peritoneal cell population obtained from patients with ascites consisting of 77% macrophages, 16% PMN leucocytes and 7% other cells (lymphocytes, eosinophils) a marked production of 6-keto-$PGF_{1\alpha}$ and 12-hydroxy-5,8,10-heptadecatrienoic acid (HHT) took place, whereas hardly any metabolites of the cyclooxygenase pathway, but mainly products of lipoxygenase, such as $LTB_4$ and 5-hydroxy-eicosatetraenoic acid (5-HETE) were observable with a highly purified peritoneal macrophage population (Fig. 4).[19,20] It is thus conceivable that the increased 6-keto-$PGF_{1\alpha}$:$TxB_2$ and 6-keto-$PGF_{1\alpha}$:$LTB_4$ ratios in the first series of experiments reflected the effect of carnitine and/or its congeners on PMN leucocytes rather than on macrophages.

L-carnitine was shown *in vitro* to exert an inhibitory influence on chemiluminescence in phorbol-myristate-acetate stimulated human PMN leucocytes.[12] Chemiluminescence is under the inhibitory control of those prostaglandins which exert their effect through enhanced levels of cyclic AMP via activation of the adenylate cyclase

**Table 1.** Effect of feeding of carnitine or its congeners on the basal and A23187 stimulated release of $LTB_4$ (ng/2 × $10^6$ macrophages)from peritoneal cells. Values are means ± SEM

| Treatment | Control | Carnitine | Acetyl carnitine | Propionyl carnitine |
|---|---|---|---|---|
| **Neutrophil contamination** | | | | |
| Basal | 0.07 ± 0.01 | 0.10 ± 0.03* | 0.14 ± 0.06* | 0.12 ± 0.04* |
| +A23187 | 0.84 ± 0.24 | 1.64 ± 0.38* | 1.86 ± 0.32* | 1.31 ± 0.31* |
| **Without neutrophil contamination** | | | | |
| Basal | 0.41 ± 0.06 | 0.36 ± 0.04 | 0.30 ± 0.03 | 0.30 ± 0.03 |
| +A23187 | 3.77 ± 1.11 | 1.86 ± 0.30 | 3.49 ± 1.12 | 2.67 ± 0.59 |

**Table 2.** Effect of feeding of carnitine or its congeners on the 6-keto-$PGF_{1\alpha}$:$TxB_2$and 6-keto-$PGF_{1\alpha}$:$LTB_4$ ratios of the basal and A23187 stimulated release of the eicosanoids from peritoneal cells. Values are means ± SEM

| Treatment | Control | Carnitine | Acetyl carnitine | Propionyl carnitine |
|---|---|---|---|---|
| **Neutrophil contamination** | | | | |
| 6kPG$F_{1\alpha}$:$TxB_2$ | | | | |
| Basal | 0.22 ± 0.01 | 0.86 ± 0.12* | 1.57 ± 0.22* | 1.00 ± 0.36* |
| +A23187 | 1.02 ± 0.21 | 4.95 ± 2.33* | 1.36 ± 0.23 | 0.82 ± 0.15 |
| 6kPG$F_{1\alpha}$:$LTB_4$ | | | | |
| Basal | 21.35 ± 2.29 | 24.70 ± 7.21 | 45.40 ± 25.43 | 24.40 ± 6.09 |
| +A23187 | 6.70 ± 1.65 | 15.19 ± 4.10* | 10.78 ± 2.93 | 9.14 ± 2.22 |
| **Without neutrophil contamination** | | | | |
| 6kPG$F_{1\alpha}$:$TxB_2$ | | | | |
| Basal | 0.48 ± 0.06 | 0.31 ± 0.03* | 0.38 ± 0.06 | 0.62 ± 0.09 |
| +A23187 | 0.90 ± 0.23 | 0.22 ± 0.04* | 0.42 ± 0.13 | 0.33 ± 0.04* |
| 6kPG$F_{1\alpha}$:$LTB_4$ | | | | |
| Basal | 8.47 ± 1.14 | 5.76 ± 0.56 | 7.62 ± 0.96 | 11.41 ± 1.82 |
| +A23187 | 6.03 ± 1.42 | 2.18 ± 0.44* | 4.06 ± 0.86 | 3.38 ± 0.61 |

complex. Macrophages obtained from renal patients on continuous ambulatory peritoneal dialysis (CAPD) during an episode of infectious dialysis show a decrease in cyclic AMP and $PGE_2$ production and an increase in TNFα and interleukin 1β (IL-1β). A cyclic nucleotide mediated influence of $PGE_2$ is recognized in the regulation of the production of TNFα from macrophages.[21,22] TNFα

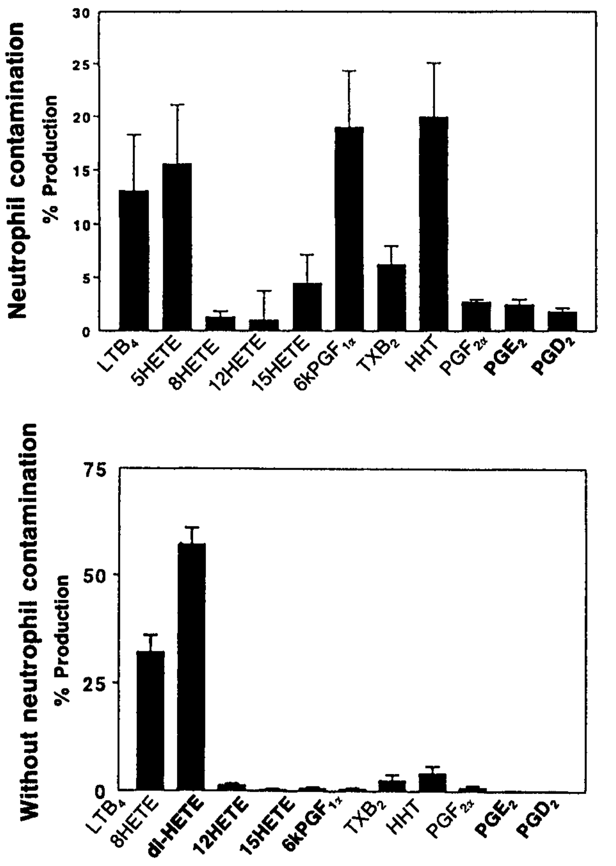

FIG. 4. Arachidonic acid metabolites formed by peritoneal cells of patients with ascites, expressed as the mean percentage of total formation of the most common metabolites ± SEM with or without neutrophil contamination.

synthesis in peritoneal rat macrophages is up-regulated by cGMP and down-regulated by cAMP, which indicates that cyclic nucleotides act as intracellular messengers for extracellular signals of macrophage activation. Whether increased production of prostaglandins and subsequently elevated levels of cyclic AMP are involved in the effect of L-carnitine on PMN leucocytes still needs to be examined. Enhanced production of prostaglandins in PMN leucocytes might have, via an interaction with concomitantly present macrophages, implications for a conceivable influence of L-carnitine or its congeners on the release of TNFα. However we were unable to observe that TNFα formation was significantly influenced by carnitine and its congeners both in rested and stimulated macrophages (data not shown). From these negative findings one could conclude that (i) the treatment regime was inappropriate to influence the release of mediators of inflammation; (ii) in this type of inflammation a clear interrelationship between $PGE_2$ and TNFα does not exist; and (iii) the *in vitro* effects of carnitine and its congeners on macrophages are not representative for the *in vivo* situation, in which the involvement of polymorphonuclear cells and their production of prostaglandins might be of importance for the discharge of TNFα from macrophages.

### References

1. Idell-Wenger JA, Neely JR. Regulation of uptake and metabolism of fatty acids by muscle. In: Dietsch JM, Gotto AM, Ontko JA, eds. *Disturbance in lipid and lipoprotein metabolism*. Am Physiol Soc Bethesda, MD, 1978; 269–284.
2. McGarry J, Rebles-Valdes C, Foster D. Role of carnitine in hepatic ketogenesis. *Proc Natl Acad Sci (USA)* 1975; **72**: 4385–4388.
3. Stryer L. *Biochemistry*. New York: W.H. Freeman and Company, 1975; 322, 472–475, 484.
4. Conte A, Fraticelli G, Ronca G. Recent findings on the regulatory functions of CoA and the normalizing activity on plasma lipids of exogeneous CoA. *Drugs Exptl Clin Res* 1992; **18**: 179–188.
5. Schepers L, *et al.* β-Oxidation of the carboxyl side chain of prostaglandin $E_2$ in rat liver peroxisomes and mitochondria. *J Biol Chem* 1988; **263**: 2724–2731.
6. Yamaoka A, Suminoto H, Isobe R, Minakami S. Formation of leukotriene $B_4$-coenzyme A ester by rat liver microsomes. *Biochem Biophys Res Comm* 1988; **154**: 1248–1252.

7. Brown GP, Monick MM, Hunninghake GW. Human alveolar macrophage arachidonic acid metabolism. *Am J Physiol* 1988; **254**: 809–815.
8. Renz H, Gong JH, Schmidt A, Nain M, Gemsa D. Release of tumour necrosis factor-alpha from macrophages. Enhancement and suppression are dose-dependent regulated by prostaglandin $E_2$ and cyclic nucleotides. *J Immunol* 1988; **141**: 2388–2393.
9. Lehmmann V, Benninghoff B, Droge W. Tumour necrosis factor-induced activation of peritoneal macrophages is regulated by prostaglandin $E_2$ and cAMP. *J Immunol* 1988; **18**: 957–959.
10. Christopherson BO, Norseth J. Arachidonic acid synthesis studied in isolated liver cells. Effects of (−)-carnitine and of (+)-decanoylcarnitine. *FEBS letters* 1981; **133**: 201–204.
11. Murakami R, Momota T, Yoshiya K, Yoshikawa N, Nakamura H, Honda M, Ito H. Serum carnitine and nutritional status in children treated with continuous ambulatory peritoneal dialysis. *J Pediatr Gastroenterol Nutr* 1990; **11**: 371–374.
12. Schinetti ML, Mazzini A. Effect of L-carnitine on human neutrophil activity. *Int J Tiss Reac* 1986; **8**: 199–203.
13. Scholte HR, *et al.* Primary carnitine deficiency. *J Clin Chem Clin Biochem* 1990; **28**: 351–357.
14. Wanner C, Riegel W, Schaefer RM, Horl WH. Carnitine and carnitine esters in acute renal failure. *Nephrol Dial Transplant* 1989; **4**: 951–956.
15. Scholte HR, Rodrigues Pereira R, Busch HF, Jennekens FG, Luyt-Houwen IE, Vaandrager-Verduin MH. Carnitine deficiency, mitochondrial dysfunction and the heart. Identical defect of oxidative phosphorylation in muscle mitochondrial in cardiomyopathy due to carnitine loss and in Duchenne muscular dystrophy. *Wien Klin Wochenschr* 1989; **6**: 12–17.
16. Elliott GR, Lauwen APM, Bonta IL. The effect of acute feeding of carnitine, acetyl-carnitine and propionyl-carnitine on basal and A23187-stimulated eicosanoid release from rat carrageenin-elicited peritoneal macrophages. *Br J Nutr* 1990; **64**: 497–503.
17. Zijlstra FJ, Vincent JE. Determination of leukotrienes and prostaglandins in [$^{14}$C]-arachidonic acid labelled human lung tissue by high-performance liquid chromatography and radioimmunoassay. *J Chrom* 1984; **311**: 39–50.
18. Garrelds IM, Zijlstra FJ, Tak CJAM, Bonta IL, Beckmann I, Ben-Efraim S. A comparison between two methods for measuring tumor necrosis factor in biological fluids. *Agents and Actions* 1993; in press.
19. Pruimboom WM, van Dijk APM, Zijlstra FJ, Wilson PJH. Effect of a novel 5-lipoxygenase inhibitor, E6080 on the eicosanoid production of human peritoneal cells. In: Nigam S. ed. *Proceedings of 2$^{nd}$ International Conference on eicosanoids and other bioactive lipids in cancer, inflammation and radiation injury.* 1993, in press.
20. Pruimboom WM. Production of inflammatory mediators by human peritoneal macrophages obtained from peritoneal ascites, in press.
21. Renz H, Gong JH, Schmidt A, Nain M, Gemsa D. Release of tumour necrosis factor $\alpha$ from macrophages. Enhancement and suppression are dose-dependent regulated by prostaglandin $E_2$ and cyclic nucleotides. *J Immunol* 1988; **141**: 2388–2393.
22. Fieren MWJA, Bemd van den GJCM, Ben-Efraim S, Bonta IL. Prostaglandin $E_2$ inhibits the release of tumor necrosis factor-$\alpha$ rather than interleukin 1$\beta$, from human macrophages. *Immunol Letters* 1991; **31**: 85–90.
